# WDR74 promotes proliferation and metastasis in colorectal cancer cells through regulating the Wnt/β-catenin signaling pathway

**DOI:** 10.1515/biol-2021-0096

**Published:** 2021-09-06

**Authors:** Zhou Cai, Yan Mei, Xiaoye Jiang, Xingfeng Shi

**Affiliations:** Department of Medical, Wuhan City College, Wuhan City, Hubei Province, 430083, China; Department of Colorectal Surgery, The Ninth People’s Hospital of Chongqing, No. 69, Jialing Village, Beibei District, Chongqing City, 400700, China

**Keywords:** WDR74, colorectal cancer, Wnt signaling, β-catenin

## Abstract

Colon cancer (CRC) is a common type of cancer and has a high incidence worldwide. Protein 74 (WDR74), which consists of the WD repetition sequence, has been previously associated with tumor tumorigenesis. However, its mechanism of action in CRC remains unclear. Here, we found that WDR74 expression was upregulated in CRC tissues and cells. Downregulation of WDR74 repressed the proliferation and cell cycles in CRC cells. In addition, WDR74 knockdown induced cell apoptosis and suppressed both cell metastasis and invasion. Mechanistically, WDR74 decreased the phosphorylation of β-catenin and induced nuclear β-catenin accumulation, activating the Wnt/β-catenin signaling pathway in CRC cells. Further investigation showed that blocking the Wnt/β-catenin signaling pathway by XAV-939 reversed the effects of WDR74 on cell proliferation, migration, and invasion in HCT116 cells. Overall, WDR74 induced β-catenin translocation to the nucleus and activated the Wnt/β-Catenin, thus facilitated CRC cell proliferation and metastasis. In summary, WDR74 could be a potential target for the intervention of CRC.

## 1 Introduction

As one of the most common gastrointestinal tumors in China and worldwide, colon cancer (CRC) has a high incidence rate and is a serious harm to people’s live and health [1,2]. With the development of medical technology, a variety of methods are used in the treatment of CRC, including surgery, radiotherapy, chemotherapy, and biological therapy; however, the 5-year survival rate of patients in an advanced state is still less than 10% [3,4]. Therefore, it is of utmost importance to identify novel and efficient anti-CRC drugs.

The Wnt/β-catenin signaling has been highly conserved in evolution and can play a role in the growth, development, tissue regeneration, occurrence of tumors and diseases, and many other physiological and pathological processes [5]. Most cases of CRC are caused by mutations in components of the Wnt signaling mutations [6]. Tanaka et al. found that β-catenin in CRC cells accumulates in the nucleus, increasing the stemless characteristics of cancer cells and the repair of DNA double-strand breaks, thereby inducing CRC cells to be sensitive to radiotherapy [7]. In addition, dysregulation of the Wnt/β-catenin signaling pathway has been shown to be related to the metastasis in CRC [8]. Moreover, oncogenic mutations in CRC result in the stabilization and translocation of β-catenin to the nucleus and induced its downstream genes expression, such as c-Myc, CCND1, and MMP-7, which contributed to cell proliferation, survival, and metastasis [9,10]. Thus, Wnt/β-catenin signaling plays a critical role in the development and progression of CRC.

Protein 74 (WDR74), consisting of the WD repetition sequence, is a central regulator of cell proliferation and embryonic development [11]. WDR74 is a 60S ribosome assembly factor, which is irreplaceable in ribosome biogenesis and is closely related to protein synthesis [12,13]. WDR74 has an important relationship with tumor regulation [14]. Studies have shown that WDR74 regulates MDM2 by regulating the level of RPL5 protein and causes the ubiquitination and degradation of p53 by MDM2, ultimately increasing the proliferation and migration of melanoma [14]. In addition, WDR74 promoted the proliferation and migration of lung cancer by inducing the nuclear translocation of β-catenin and activating the Wnt signaling pathway [15,16]. However, WDR74 has rarely been reported in CRC and its underlying mechanism of action is unclear.

This study showed that WDR74 is highly expressed in CRC cells and is involved in the proliferation and migration in CRC by regulating the Wnt/β-catenin signaling pathway. Mechanically, WDR74 induced nuclear β-catenin accumulation and repressed β-catenin phosphorylation-dependent degradation in CRC cells. Together, our findings demonstrate that WDR74 can be a potential target for the clinical treatment of CRC.

## 2 Methods

### 2.1 TCGA analysis

The gene expression of WDR74 in the Cancer Genome Atlas (TCGA) Data Portal (https://tcga-data.nci.nih.gov/tcga/) was analyzed using the UCSC Cancer Genomic Browser (https://genome-cancer.soe.ucsc.edu/proj/site/hgHeatmap/). TCGA CRC RNAseq (IlluminaHiSeq; *N* = 488) and 41 matched normal samples were used for the comparison of WDR74 expression.

### 2.2 Antibodies and reagents

Antibodies directed against β-catenin, p-β-catenin, E-cadherin, N-cadherin, Bax, Bcl, c-caspase 3, caspase 3, β-actin, and β-tubulin were purchased from Cell Signaling Technologies (Danvers, MA, USA). Antibodies directed against histone H3 were purchased from Santa Cruz Biotechnology (Santa Cruz, CA, USA). Antibodies directed against WDR74 were purchased from OriGene (Rockville, MD, USA). Antibodies directed against lef1, TCF1, c-myc, and cyclin D1 were purchased from Abisin (Shanghai, China). XAV-939(S1180), a Wnt/β-catenin signaling pathway-specific inhibitor, was purchased from Selleck Chemicals (Houston, TX, USA).

### 2.3 Cell and cell culture

CRC cells lines, HT29, HCT116, SW48, and HCT15, and human normal colon epithelial cell line (NCM460) were purchased from the Cell Resource Center of Otwo Biotech (Shenzhen) Inc (Shenzhen, China).

Cells were cultured with Dulbecco’s Modified Eagle’s Medium (DMEM) containing 10% fetal bovine serum (FBS), except for HCT116 cells, which were cultured in complete McCoy’s 5A medium. All cells were cultured in the presence of 1% penicillin and 1% streptomycin at 37°C and 5% CO_2_, respectively.

### 2.4 WDR74 silencing and overexpression in HCT116 and HT39 cells

Cells (10^5^ cells/well) were cultured in 6-well plates. Sh- RNA-targeting WDR47 and ov-WDR47 (WDR47 expressing plasmids) and corresponding control vectors, constructed by Sangon Biotech (Shanghai, China), were transfected into cells using Lipofectamine 3,000 (Invitrogen, Carlsbad, CA), following the manufacturer’s guidelines. Subsequently, the cells were cultured at 37°C and 5% CO_2_ for 36 h. Then, cells were harvested, and the transfection efficiency was determined by Western blot analysis.

### 2.5 Cell viability assay

Cell viability was determined by the CCK8 assay (Meilunbio, Dalian, China). In brief, CRC cells transfected with appropriate plasmids were seeded into 96-well plates with 10,000 cells/well, cultured at 37°C with 5% CO_2_ for 6 h, and then replaced the medium with 10% CCK8 for 2 h at 37°C. The cell viability was determined at 450 nm using the Infinite M200 Pro microplate reader (Tecan, Swiss).

### 2.6 Cell-cycle assay

First, the cells were washed twice with prechilled PBS and fixed with 70% ethanol at 4°C for 24 h. Then, cells were collected and stained with propidium iodide (PI) fluorescent stain (1% (v/v) Triton X-100; 0.01% RNase; 0.05% PI (Sigma-Aldrich)) for 30 min at 37°C, protected from light. The cell suspension was then loaded into a flow cytometer (BD) for DNA content analysis. Finally, Modfit software 3.2 was used to determine cycle distribution.

### 2.7 Cell proliferation assay

Cell proliferation was determined using the cell colony formation assay. In brief, cells (10^4^ cells/well) treated with ov-NC, ov-WDR74, sh-NC, and sh-WDR74 were seeded into 6-well plates to establish cell colonies. Fourteen days later, the cells were fixed with 4% paraformaldehyde for 30 min and stained using 0.2% crystal violet for 40 min. The overall map of cell colonies was then scanned and the number of colonies was counted.

### 2.8 Western blotting analysis

Total proteins were extracted with RIPA lysate containing 1% PMSF on ice. The extraction of nucleoproteins and cytoplasmic proteins was performed according to the kit manufacturer’s instructions (Meilunbio, Dalian, China). The extracted protein samples were diluted with 5× loading buffer and denatured at 95°C for 5 min. Then, total proteins (40–50 μg) were separated on 10% SDS-PAGE gel and transferred to polyvinylidene difluoride (PVDF) membranes. Next, membranes were blocked with 5% nonfat milk for 1 h at room temperature and incubated with primary antibodies overnight at 4°C. Then, membranes were washed with TTBS and incubated consecutively with a horseradish-peroxidase (HRP)-conjugated secondary antibody for 1.5 h at room temperature. Finally, proteins were visualized by incubation with ECL reagent.

### 2.9 Statistical analysis

Data were analyzed and processed using GraphPad Prism 7.0 and presented as the mean ± SD. At least three separate experiments were carried out per experiment. We used mean ± standard error of mean unless otherwise stated. Student’s *t*-test was used to detect significances between the groups; *p* < 0.05 was considered statistically significant.

## 3 Results

### 3.1 WDR74 was upregulated in CRC cells

**Figure 1:**
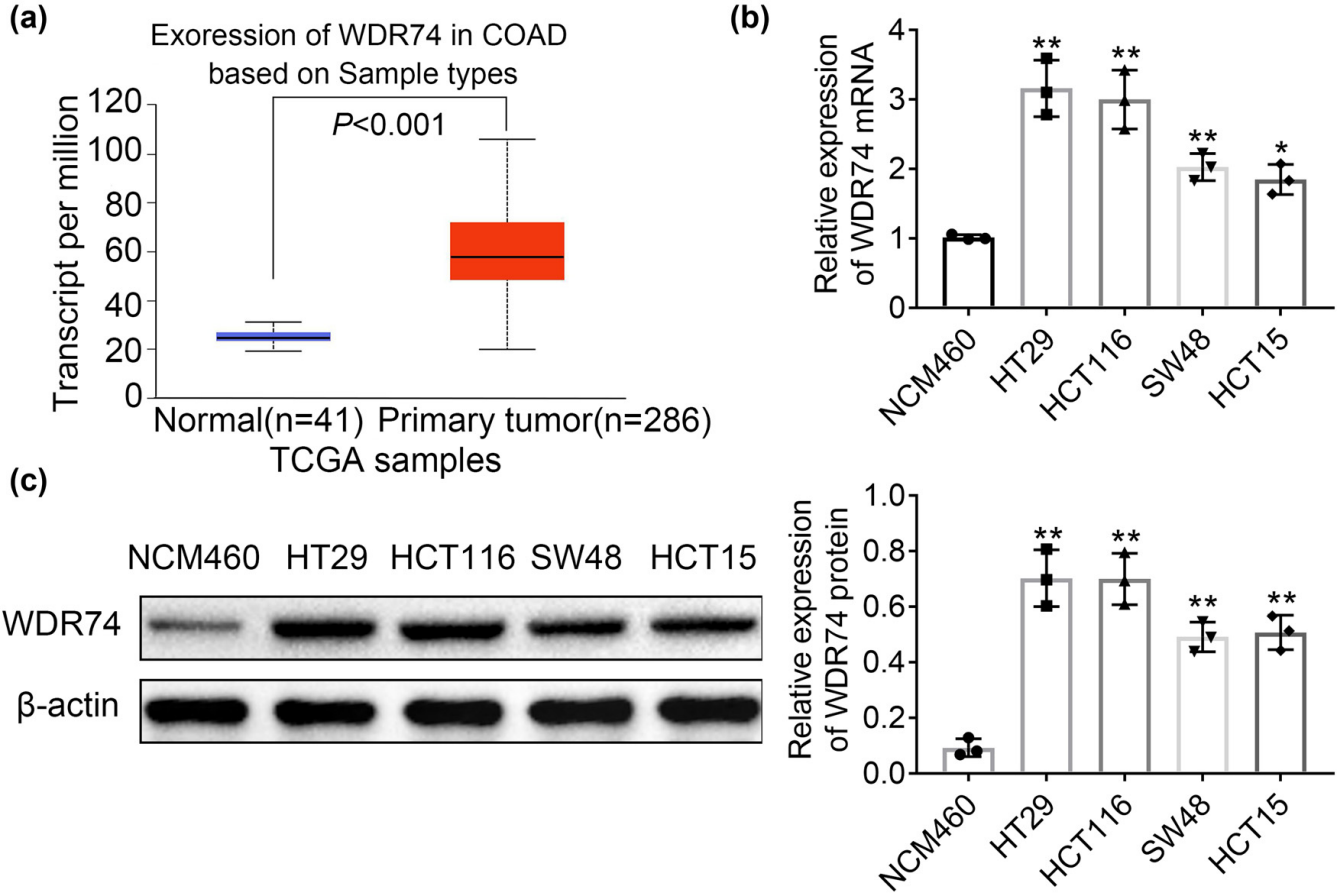
WDR74 was upregulated in CRC cells. (a) WDR74 is highly expressed in most CRC tumors (TCGA database online website GEPIA: http://gepia.cancer-pku.cn/). (b) The mRNA level of WDR74 in human normal colon epithelial cell line (NCM460) and four CRC cell lines (HT29, HCT116, SW48, HCT15). (c) The protein expression of WDR74 in human normal colon epithelial cell line (NCM460) and four CRC cell lines (HT29, HCT116, SW48, HCT15). ^*^ *p* < 0.05, compared with NCM460; ^**^ *p* < 0.01, compared with NCM460. Results are presented as mean ± SD of three independent experiments.

Previous studies have shown that WDR74 acts as a coactivator in TGF-β signaling and is involved in a variety of biological processes including cell proliferation, cell differentiation, cell signaling, and gene transcription [15]. WDR74 has been implicated to participate in the metastatic potential of cancer cells [17]. To further investigate the role of WDR74 in CRC, the expression of WDR74 was analyzed in large datasets from TCGA databases. Data showed that the expression of WDR74 was increased in 288 CRC tumor tissues when compared with that in 41 normal colorectal tissues (Figure 1a). Next, we determined the mRNA and protein levels of WDR74 in CRC cell lines HT29, HCT116, SW48, and HCT15, and the expression of WDR74 was consistently upregulated in CRC cell lines compared to controls (Figure 1b and c).

### 3.2 WDR74 depletion suppresses the proliferation of CRC cells *in vitro*

**Figure 2:**
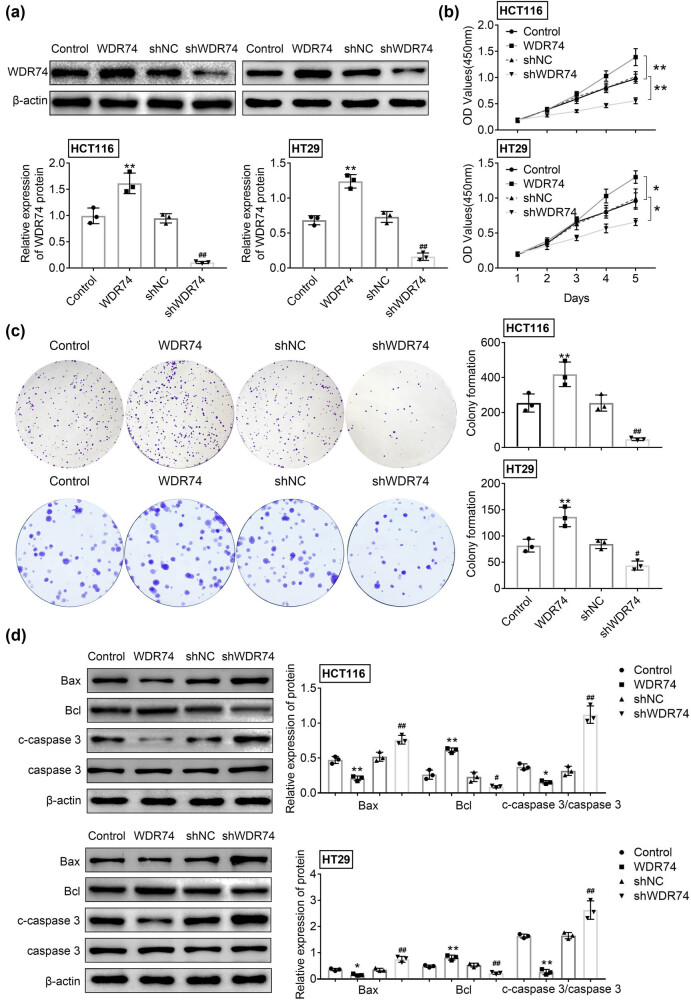
The effect of WDR74 depletion on cell proliferation in CRC cells. (a) The protein expression of WDR74 in CRC cells treated with sh-NC, sh-WDR74, ov-NC, and ov-WDR74. (b). The cell viability was assessed by CCK-8 assay. (c) Colony forming ability of CRC cells as measured by colony formation assay. (d) The expression level of Bax, Bcl, and c-caspase 3/caspase 3 were detected by western-blot. ^**^ *p* < 0.01, compared with control; ^##^ *p* < 0.01, compared with sh-NC. Results are presented as mean ± SD of three independent experiments.

To further evaluate the biological function of WDR74 expression in CRC cells, WDR74 knockdown was performed by WDR74-targeting shRNA, and WDR74 expressing plasmids were transfected into CRC cell lines HCT116 and HT29. The expression of WDR74 was confirmed by Western blot analysis to validate the transfection efficiency (Figure 2a). Absorbance assays showed a significantly decreased number of CRC cells in the sh-WDR74 group compared with cells in the control group after 5 days of culture (Figure 2b). Conversely, WDR74 overexpression significantly induced cell growth activity (Figure 2b). Our data showed enhanced cell proliferation in WDR74 expressing cells, while depletion of WDR74 in CRC cells significantly inhibited the cell colony formation ability, as indicated by colony cell numbers (Figure 2c). Moreover, we examined the effect of WDR74 on cell apoptosis in CRC cells and found that WDR74 overexpression inhibited the expression of proapoptotic proteins Bcl and C-caspase3/caspase3 and suppressed the expression of the anti-apoptotic protein Bax. These results were reversed after inhibition of WDR74 expression (Figure 2d), indicating that WDR74 knockdown can promote apoptosis in CRC cells. The above results suggest that WDR74 has an oncogenic effect on CRC cells through promoting cell growth and inhibiting cell apoptosis.

### 3.3 WDR74 knockdown inhibited the cell cycle in CRC cells

**Figure 3:**
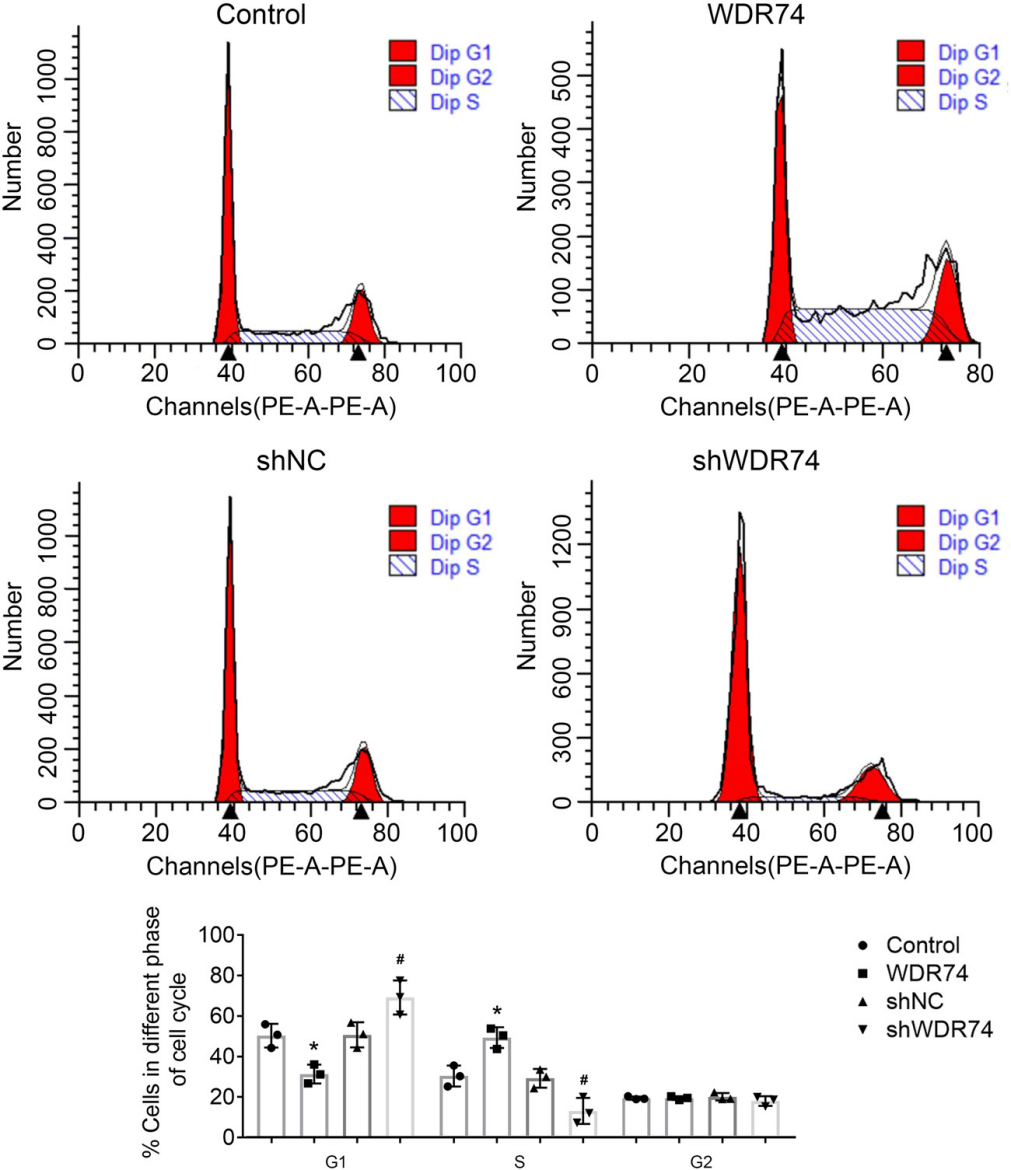
WDR74 accelerates cell-cycle progression in HCT116 cells. WDR74 knockdown decreased cell population in G1 phase and increased cell population in S-phase in HCT116 cells by using flow cytometry. ^**^ *p* < 0.01, compared with control; ^##^ *p* < 0.01, compared with sh-NC. Results are presented as mean ± SD of three independent experiments.

To further clarify the intrinsic molecular mechanism by which WDR74 promotes cell proliferation, we assessed the role of WDR74 on the cell-cycle phases using flow cytometry. Upregulated expression of WDR74 in HCT116 cells decreased the number of G0 to G1 phase cells and increased the number of S phase cells accordingly (Figure 3a and b). In addition, WDR74 knockdown in HCT116 cells induced G0 to G1 arrest (Figure 3a and b), suggesting that WDR74 may promote G1 to S transition in HCT116 cells. Thus, these results indicate that WDR74 is necessary for the malignant proliferation of CRC cells.

### 3.4 WDR74 knockdown suppressed cell migration and invasion of CRC

**Figure 4:**
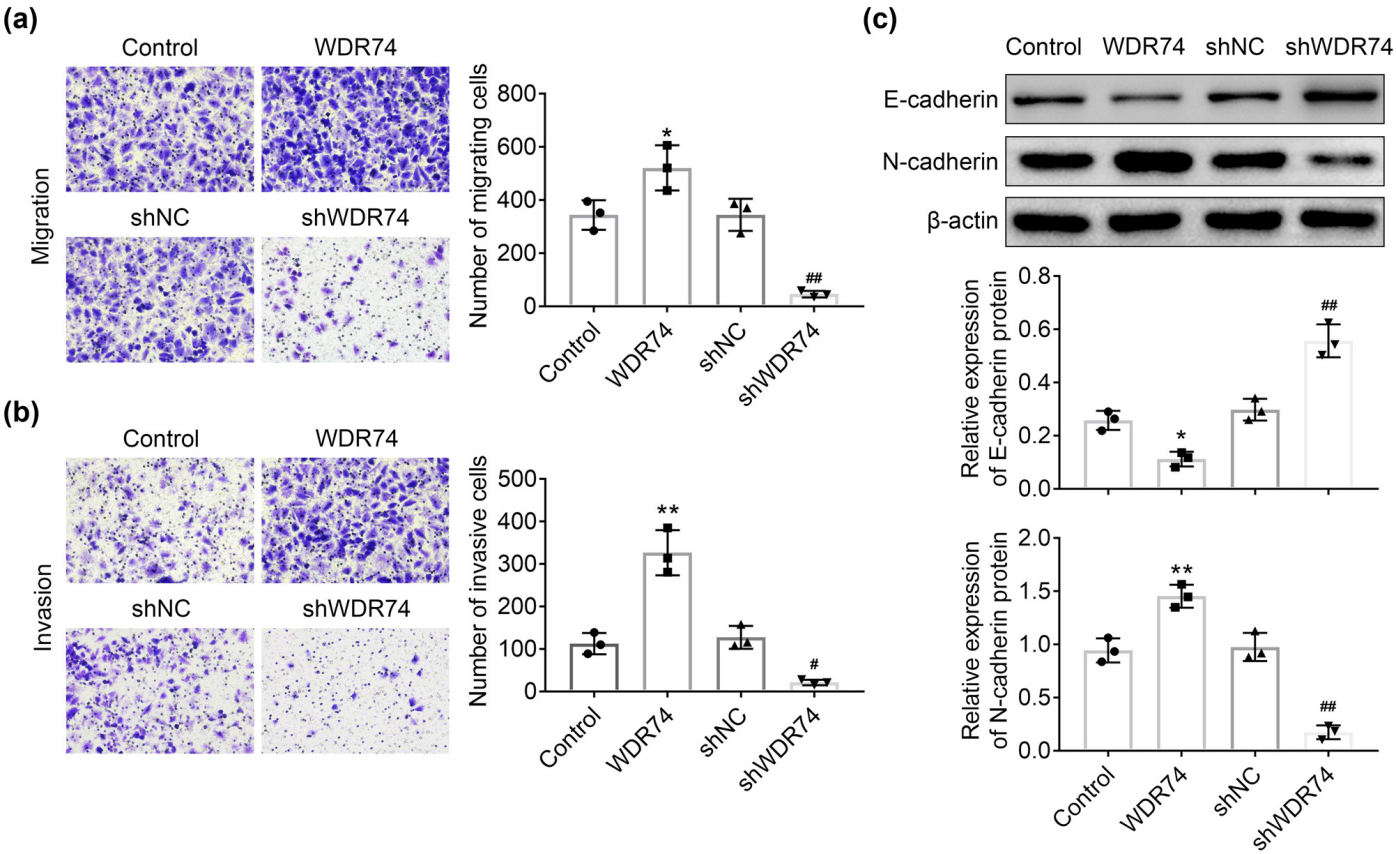
WDR74 knockdown suppressed migration and invasion of CRC cells. (a) and (b) The transwell assay in HCT116 cells transfected with sh-NC, sh-WDR74, ov-NC, and ov- WDR74. C Protein levels of EMT markers determined by western-blot in HCT116 cells. ^*^ *p* < 0.05, compared with control; ^**^ *p* < 0.01, compared with control; ^##^ *p* < 0.01, compared with sh-NC. Results are presented as mean ± SD of three independent experiments.

We next determined the effect of WDR74 on cell migration and invasion ability using Transwell assays in HCT116 cells. The results showed that the cell migration and invasion ability were significantly increased in the absence of WDR74 (Figure 4a and b). The previous studies showed that the activation of epithelial-mesenchymal transition (EMT) is essential for the metastasis of cancer cells to distant sites. Therefore, we evaluated the effect of WDR74 on the expression of EMT markers, including E-cadherin and N-cadherin. Our data showed that WDR74 expression induced N-cadherin and repressed E-cadherin expression (Figure 4c), thereby indicating the activation of EMT. Moreover, WDR74 knockout eliminated EMT activation by reversing the expression of N-cadherin and inducing the expression of E-cadherin (Figure 4c). Taken together, these findings reveal that WDR74 promotes the metastasis of CRC cells.

### 3.5 WDR74 knockdown inhibited the Wnt/β-catenin signaling pathway in CRC cells

**Figure 5:**
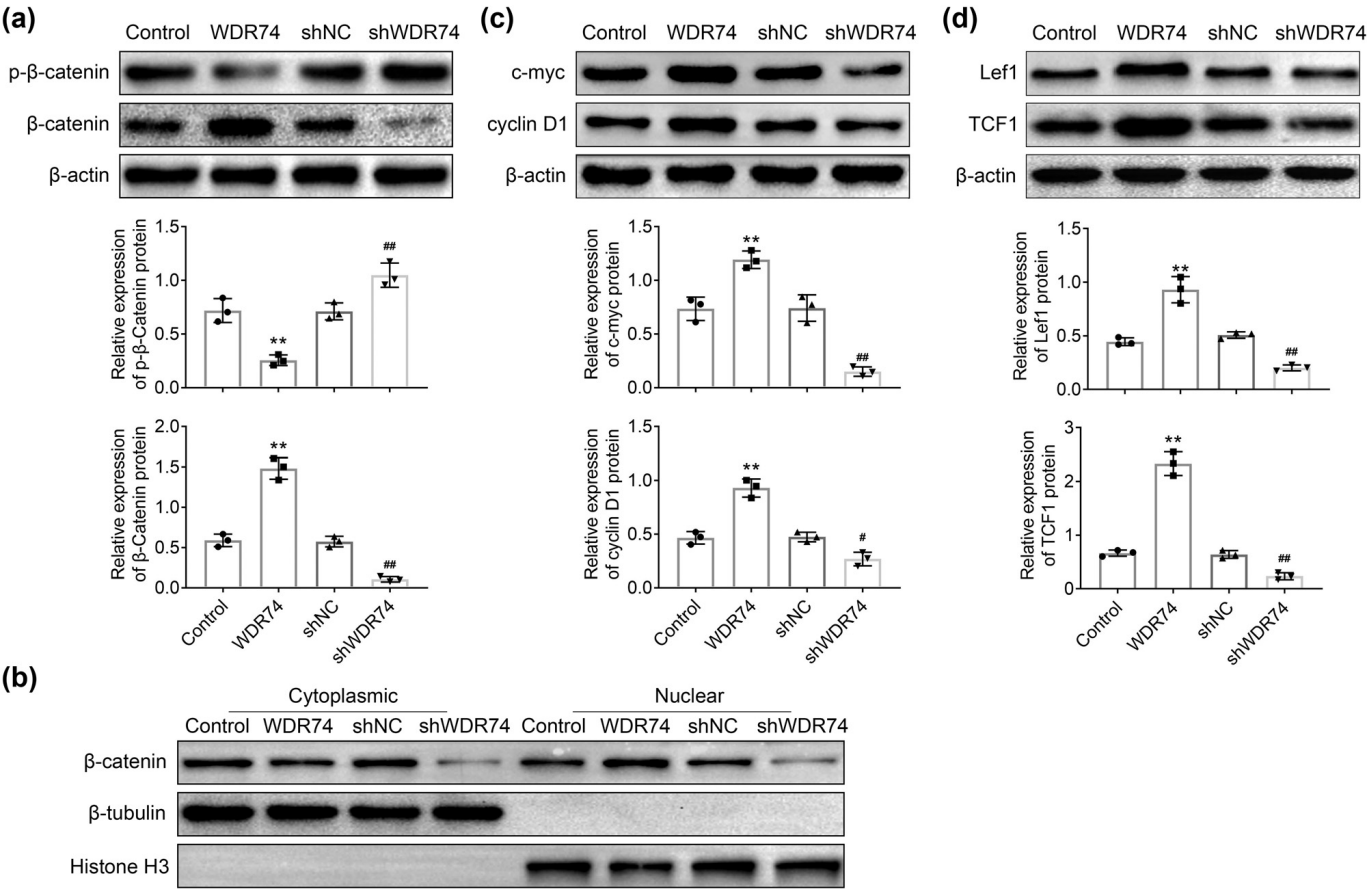
WDR74 knockdown inhibited Wnt/β-Catenin signaling pathway in CRC cells. (a) The protein expression of p-β-Catenin and β-Catenin in HCT116 cells detected by western-bolt. (b) The levels of β-Catenin in cell cytoplasmic and nuclear, respectively, in HCT116 cells treated with sh-NC, sh-WDR74, ov-NC, and ov-WDR74. (c) and (d) HCT116 cells treated with sh-NC, sh-WDR74, ov-NC, and ov-WDR74, and the expression levels of c-myc, cyclin D1, lef1, and TCF1 were detected by western-bolt. ^**^ *p* < 0.01, compared with control; ^##^ *p* < 0.01, compared with sh-NC. Results are presented as mean ± SD of three independent experiments.

We next explored whether the effect of WDR74 on CRC proliferation and invasion was mediated by Wnt/β-catenin signaling, which is well-known to regulate the development of CRC cells [18]. We found that WDR74 overexpression suppressed the phosphorylation of β-catenin, while WDR74 downregulation improved these characteristics reciprocally (Figure 5a). Furthermore, WDR74 promoted the translocation of β-catenin from the cytoplasm to the nucleus, thereby inducing the dramatic increase in the expression of nuclear β-catenin(Figure 5b). Thus, WDR74 suppresses the degradation of cytosolic β-catenin and promotes nuclear β-catenin accumulation, accompanied by the regulation of downstream target genes to affect the cell biological behavior in CRC cells (Figure 5c and d).

**Figure 6:**
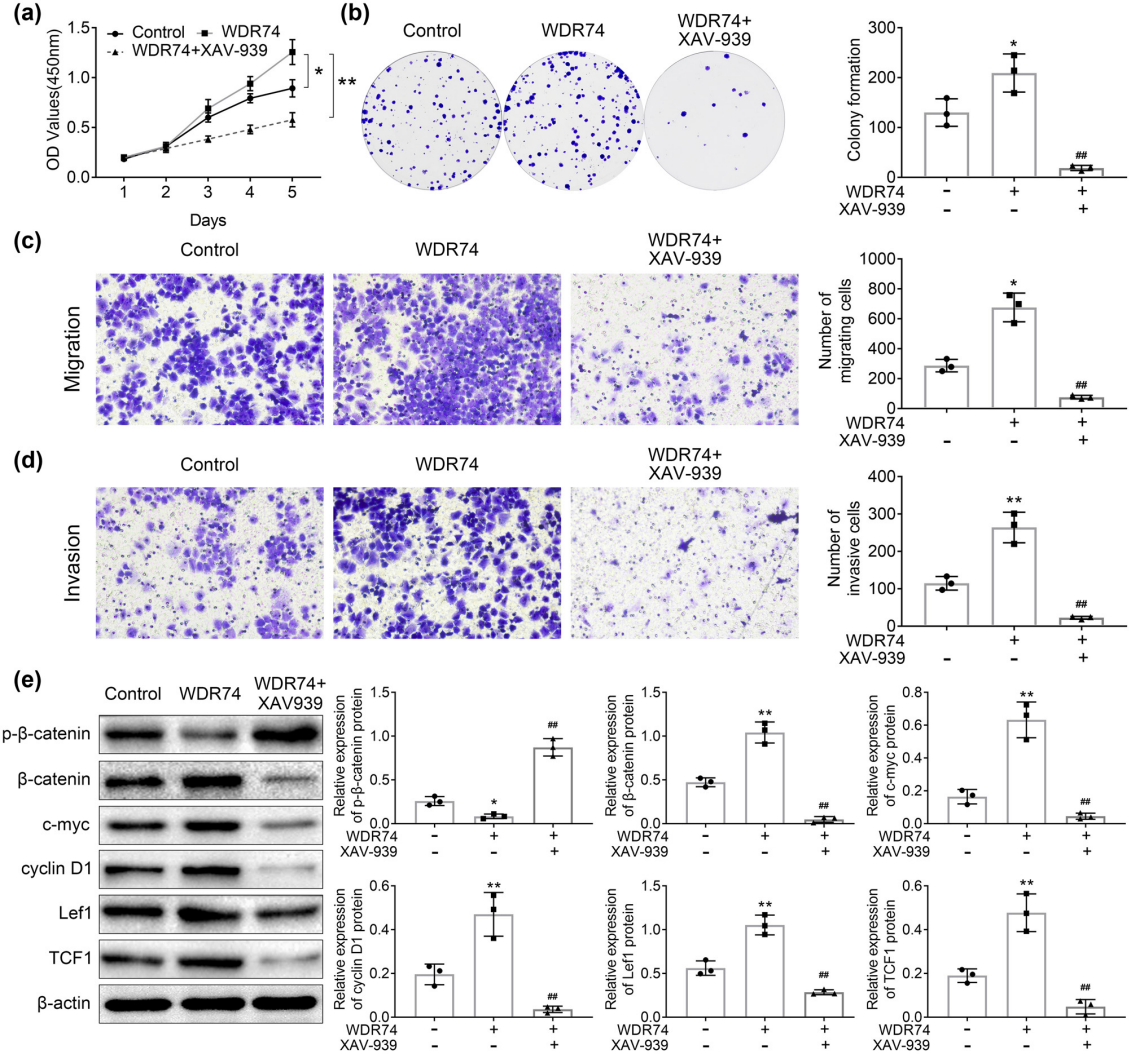
The oncogenetic effect of WDR74 was reversed by XVA-939. (a) Cell proliferation was detected by CCK8 assay after WDR74 overexpression with or without XVA-939 (1 μM). (b) Colony forming ability in HCT116 cells as measured by colony formation assay. (c) and (d) HCT116 cells transfected with ov-WDR74 with or without XVA-939(1 μM). Cell migration and invasion ability were determined by Transwell assay. (e) The protein levels of p-β-Catenin, β-Catenin, c-myc, cyclin D1, lef-1, and TCF-1. (*n* = 3) ^**^ *p* < 0.01, compared with control; ^##^ *p* < 0.01, compared with on-WDR74. Results are presented as mean ± SD of three independent experiments.

To further validate the role of WDR74 in CRC development through the Wnt/β-catenin pathway, XAV-939, a Wnt/β-catenin signaling pathway-specific inhibitor, was used in the present study. We found that XAV-939 reversed the effect of WDR74 on cell growth, migration, and invasion in HCT116 cells (Figure 6a–d). Besides, XAV939 reversed the expression levels of p-β-catenin, β-catenin, c-myc, Cyclin D1, Lef1, and TCF1, which are the targets of wnt signaling (Figure 6e). Thus, the results demonstrated that WDR74 induced CRC development via the Wnt/β-catenin pathway.

## 4 Discussion

Nowadays, with the development of science and technology, cancer research has made significant progress; however, overcoming cancer is still one of the biggest challenges. CRC is one of the most prevalent cancers worldwide and has a high incidence [19]. Early-onset CRC can be easily eradicated by surgery; however, its treatment is challenging for patients with metastases or postoperative recurrence [20]. In this study, we focused on the regulation of CRC progression and metastasis through WDR74. We investigated the oncogenic role of WDR74 in CRC and found a significant increase in WDR74 expression in CRC cells, which was corroborated with the results from the TCGA database in CRC tumor samples (Figure 1). WDR74 has been shown to be a novel Smad-binding protein associated with the pathogenesis of lung cancer [15,16]. However, the underlying molecular mechanism of WDR74 in the management of CRC development remains unclear.

In this study, we first found that WDR74 is involved in the growth and progression of CRC cells. The upregulation of WDR74 expression was related to cell growth, cell colony formation, and cell-cycle progression. To explore the carcinogenesis role of WDR74 in CRC cells, we constructed shWDR74 to reduce the expression level of WDR74 in CRC cells. WDR74 silencing significantly inhibited cell growth (Figure 2a and b), cell colony formation (Figure 2c), and cell-cycle progression (Figure 3). Moreover, WDR74 downregulation induced cell apoptosis (Figure 2d). Importantly, WDR74 knockout damaged cell invasion and metastasis behaviors and blocked the EMT activation (Figure 4). Hence, these results suggest that WDR74 acts as an effective regulator in the progression of CRC.

Wnt/β-catenin signaling plays a significant role in maintaining development and homeostasis in a variety of tissues throughout the body [21]. In addition, in previous studies, it has been shown that Wnt/β-catenin signaling plays a critical role in CRC initiation and early progression, as well as in late invasion and metastasis [22,23]. In the present study, we demonstrated that downregulation of WDR74 decreased β-catenin expression in the nucleus and promoted phosphorylation-dependent degradation of β-catenin (Figure 5). The β-catenin translocation from the cytoplasm to the nucleus is the primary step in the activation of the Wnt/β-catenin signaling pathway [24]. Thus, WDR74 promotes the malignant transformation of CRC cells associated with the nuclear localization of β-catenin. Subsequently, the accumulated β-catenin in the nucleus further induced the expression of Wnt-related genes involved in cell proliferation and apoptosis [25,26]. Moreover, WDR74 expression also affected the expression of Wnt/β-catenin downstream genes, such as lef1, TCF1, c-myc, and cyclin D1, which have been implicated in cell proliferation [27,28], and caspase 3 is related to cell apoptosis [29]. WDR74 knockout decreased the expression of lef1, TCF1, c-myc, and cyclin D1 (Figure 5c and d) and induced the expression of caspase 3 (Figure 2d). Pharmacological inhibition of the Wnt/β-catenin signaling revered the effect of WDR74 overexpression on CRC cells, as well as the regulation of Wnt/β-catenin pathway (Figure 6). Thus, these findings highlight the potential role of WDR74 and its regulation of the Wnt/β-catenin signaling pathway in CRC cells. However, further studies and *in vivo* validation are required in the future.

In this study, we first demonstrated the molecular mechanism of WDR74 in CRC and its interaction with cancer proliferation and metastasis. Furthermore, we showed that WDR74 plays an important role in the regulation of Wnt/β-catenin signaling. These results collectively provide significant mechanistic progress into the growth and metastasis of CRC cells and indicated that the WDR74 server is a therapeutic and diagnostic target for CRC remedy.
